# Peripheral Axonal Neuropathy in Hemophagocytic Lymphohistiocytosis Secondary to Rickettsia conorii Infection

**DOI:** 10.7759/cureus.75598

**Published:** 2024-12-12

**Authors:** Adriana Henriques, Mariana Guerra, Ana Rita Ramalho, Ana Coimbra, Jandira Lima

**Affiliations:** 1 Internal Medicine, Centro Hospitalar e Universitário de Coimbra, Coimbra, PRT

**Keywords:** adrenal cortex hormones, hemophagocytic lymphohistiocytosis, hemophagocytic syndrome infection-associated, polyneuropathy, rickettsia conorii

## Abstract

Hemophagocytic lymphohistiocytosis (HLH) is a rare and potentially fatal hyperinflammatory syndrome characterized by dysregulated immune activation and systemic inflammation. Secondary HLH is often triggered by infections, with *Rickettsia conorii* being an infrequently reported cause. Peripheral axonal neuropathy is a rare and poorly understood complication of HLH. We present the case of a 58-year-old male who presented with fever, diarrhea, a maculopapular rash involving the palms and soles, and progressive lower limb weakness. Laboratory findings revealed pancytopenia, hyperferritinemia, elevated soluble CD25, liver cytolysis, hypofibrinogenemia, and raised inflammatory markers. Serological testing confirmed *Rickettsia conorii *infection. The patient fulfilled six of the eight HLH-2004 diagnostic criteria, establishing the diagnosis of secondary HLH. Treatment was initiated with intravenous dexamethasone and doxycycline. During hospitalization, the patient developed severe hypoxemic respiratory failure, paraplegia, and acute sensory loss. The neurological evaluation confirmed peripheral axonal neuropathy linked to *Rickettsia conorii *infection, which preceded the development of HLH. After 21 days of doxycycline therapy, the patient was discharged to a rehabilitation facility with full neurological recovery. This case underscores the rarity of *Rickettsia conorii* as a trigger for HLH and highlights the exceptional occurrence of peripheral axonal neuropathy as a complication. The singularity of this case reinforces the importance of a correct and early diagnosis of HLH, which can have atypical and severe complications but also a successful outcome.

## Introduction

Hemophagocytic lymphohistiocytosis (HLH) is a rare and potentially fatal disease characterized by an acute, rapidly progressive systemic hyperinflammatory response [1]. This condition results from excessive activation of cytotoxic T lymphocytes and histiocytes and generalized activation of macrophages that phagocytize healthy hematopoietic cells. This mechanism triggers a failure in the inhibitory control of cytotoxic T lymphocytes, leading to overproduction of pro-inflammatory cytokines and systemic accumulation of activated T cells and macrophages, perpetuating a hyperinflammatory state [1,2].

Although predominantly a pediatric condition, HLH can occur at any age [2]. It can be hereditary or acquired, being, in this case, triggered by infections, malignant tumors, autoimmune diseases, or immunosuppression [3].

Among infectious causes, viral infections are most common in adults, particularly Epstein-Barr virus*.* Nevertheless, bacterial agents, such as *Rickettsia conorii, *can also be precursors. The spectrum of clinical manifestations is broad, including persistent fever, hepatosplenomegaly, pancytopenia, hypertriglyceridemia, hypofibrinogenemia, hyperferritinemia (>500 mcg/L), elevated soluble CD25, and absence or reduced activity of natural killer (NK) cells.

Despite being a pathognomonic finding for the diagnosis of HLH, the presence of hemophagocytosis in bone marrow, liver, or lymph node samples is not essential for the assumption of the disease. Diagnostic criteria such as those established in the HLH-2004 protocol remain widely used in clinical practice [4,5].

Neurological involvement is known to be present in up to 30% of published cases, including encephalitis, seizures, ataxia, and behavioral changes [6]. Peripheral axonal neuropathy, as documented in this case, is an extremely rare complication, possibly resulting from macrophage-mediated destruction of the myelin sheath, leading to significant motor and/or sensory sequelae [7].

In this context, we report a case of HLH secondary to infection with *Rickettsia *spp., presenting with peripheral axonal neuropathy, contributing to the limited literature on neurological and systemic complications of this rare condition.

## Case presentation

We present the case of a 58-year-old male admitted with a one-day history of diarrhea, fever, and a generalized maculopapular rash involving the palms and soles. The patient also reported progressive muscle weakness in the lower limbs that had started 10 days before admission. He denied other complaints. His medical history included type 1 diabetes mellitus, medicated with human isophane insulin (8 units for breakfast and 6 units for dinner), with poor compliance to treatment and poor metabolic control of the disease. He also had an alcohol use disorder, lived in a rural area, and had a dog as his only pet.

On initial physical examination, the patient was disoriented, hypotensive (BP 70/40 mmHg), tachycardic (HR 101 bpm), and febrile (39°C). A maculopapular rash with scattered petechiae was noted on the trunk, limbs, palms, and soles without signs of inoculation lesions ("tache noire"). Neurological examination revealed isochoric and reactive pupils, no deficits in ocular movements or visual fields, no facial asymmetry or dysmetria, normal language function, tongue midline alignment, and no motor strength deficits in the upper and lower limbs. Sensory, coordination, and gait assessments were unremarkable.

Initial laboratory tests (Table 1) showed elevated inflammatory markers (C-reactive protein (CRP) 8.52 mg/dL, procalcitonin 2.39 ng/mL), liver cytolysis (alanine aminotransferase (AST) 985 U/L, alanine aminotransferase (ALT) 175 U/L), elevated cholestatic enzymes (gamma-glutamyl transferase (GGT) 344 U/L, ALP 165 U/L), pancytopenia (leukocytes 2.7x10^9^/L, hemoglobin 10.7 g/dL, platelets 67x10^9^/L), hypofibrinogenemia (39 mg/dL), hyperferritinemia (65,865 ng/mL), and elevated soluble CD25 (3,124 U/mL). Arterial blood gas analysis identified hyperlactatemia (2.6 mmol/L).

**Table 1:** Analytical results at admission LDH: lactate dehydrogenase; AST: aspartate aminotransferase; ALT: alanine aminotransferase; GGT: gamma-glutamyl transferase; MCV: mean corpuscular volume

Serum Analysis	Emergency Department	Reference Values
LDH	670 U/L	<248 U/L
AST	985 U/L	<31 U/L
ALT	175 U/L	<34 U/L
GGT	344 U/L	<38 U/L
Alkaline phosphatase	165 U/L	30-120 U/L
Total bilirubin	1.7 mg/dL	0.2-1.2 mg/dL
Direct bilirubin	1.3 mg/dL	<0.5 mg/dL
Albumin	2.3 g/dL	3.5-5.2 g/dL
C-reactive protein	8.52 mg/dL	<0.5 mg/dL
Procalcitonin	2.39 ng/mL	0-0.5 ng/mL
Leukocytes	2.7x10^9^/L	4.0-10.0x10^9^/L
Hemoglobin	10.7 g/dL	12-15.6 g/dL
MCV	107 fL	80-99 fL
Platelets	67.0x10^9^/L	150-400x10^9^/L
Prothrombin time	57%	70-120%
Fibrinogen	39 mg/dL	200-500 mg/dL
Ferritin	65,865 ng/mL	30-300 ng/mL
Triglycerides	277 mg/dL	43.8-195.1 mg/dL
Soluble CD25	3,124 U/mL	-

**Figure 1:**
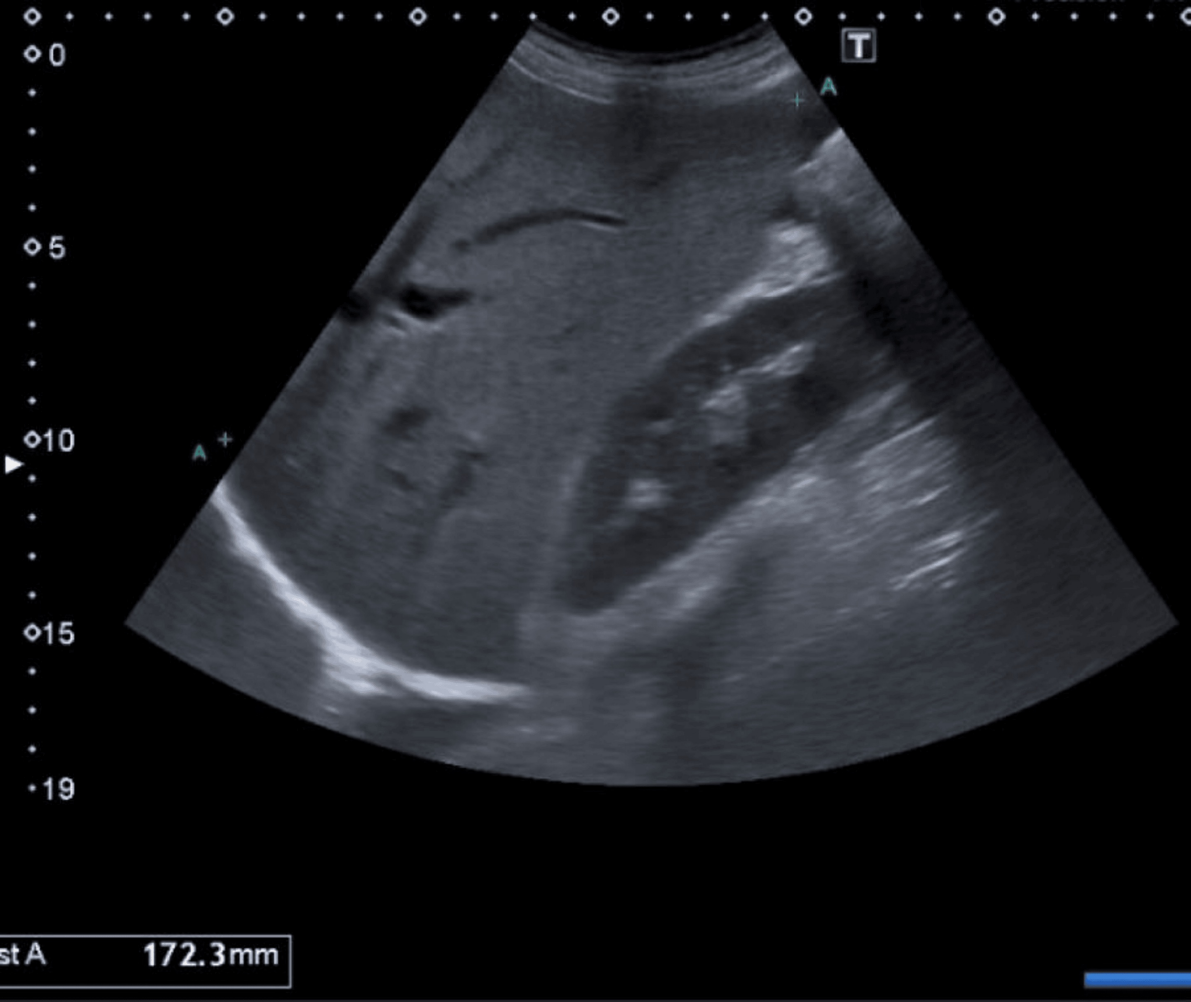
Hepatomegaly detected on abdominal ultrasound

An abdominal ultrasound revealed hepatomegaly (17.2 cm) (Figure 1) with a hyperechoic structure suggestive of steatosis. The spleen was of normal size and homogeneous.

Septic screening, including blood and urine cultures, was negative. Infectious serologies were collected, but results were pending. Empirical treatment with ceftriaxone (2 g/day) and intravenous dexamethasone (10 mg/m^2^/day) was initiated based on suspected HLH secondary to an undetermined infectious process.

On the third day of hospitalization, the patient experienced a sudden worsening of consciousness and new-onset hypoxemic respiratory failure (PaO_2_/FiO_2_ ratio 80), requiring invasive mechanical ventilation.

**Figure 2:**
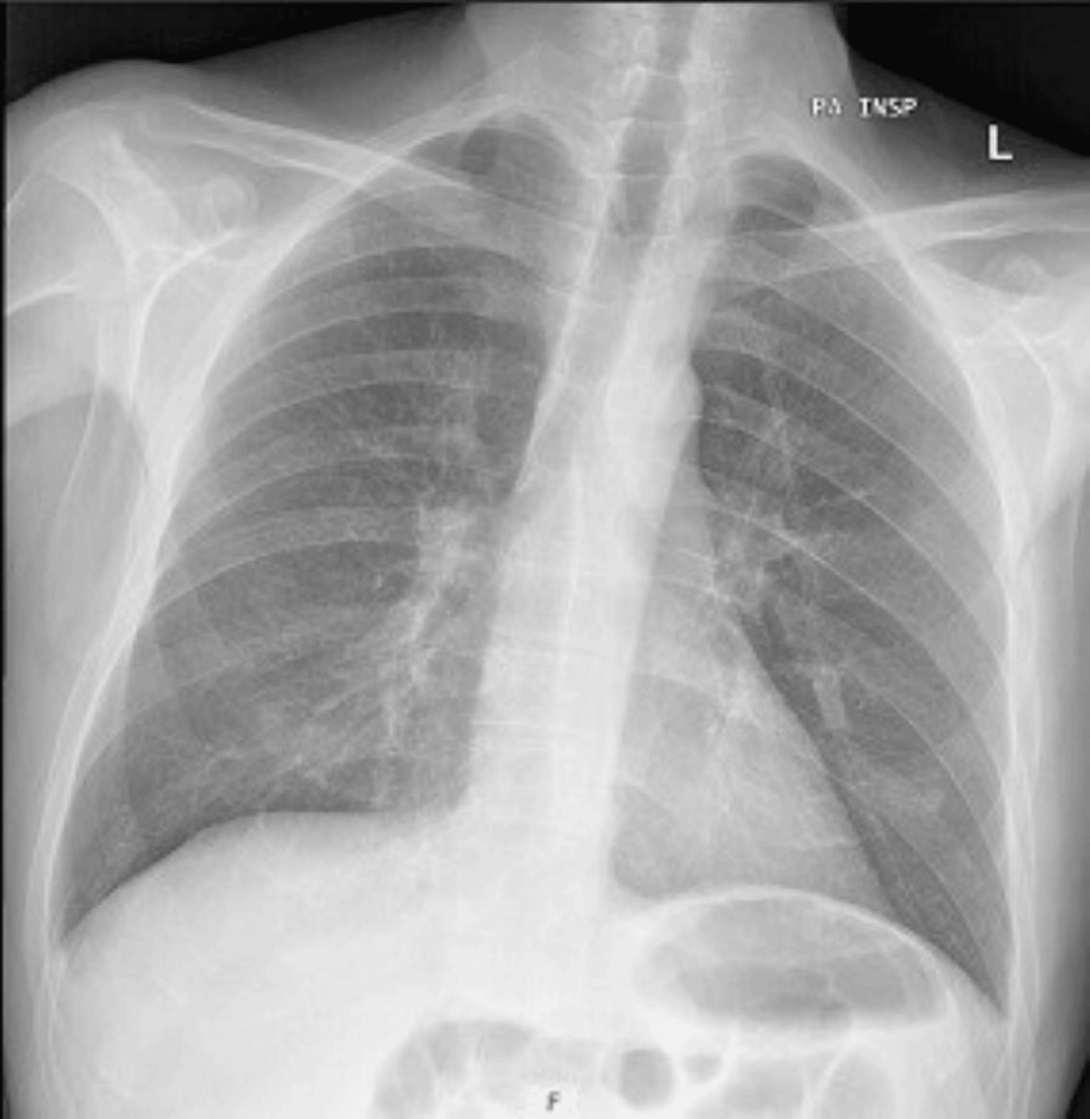
Chest X-ray without significant alterations

**Figure 3:**
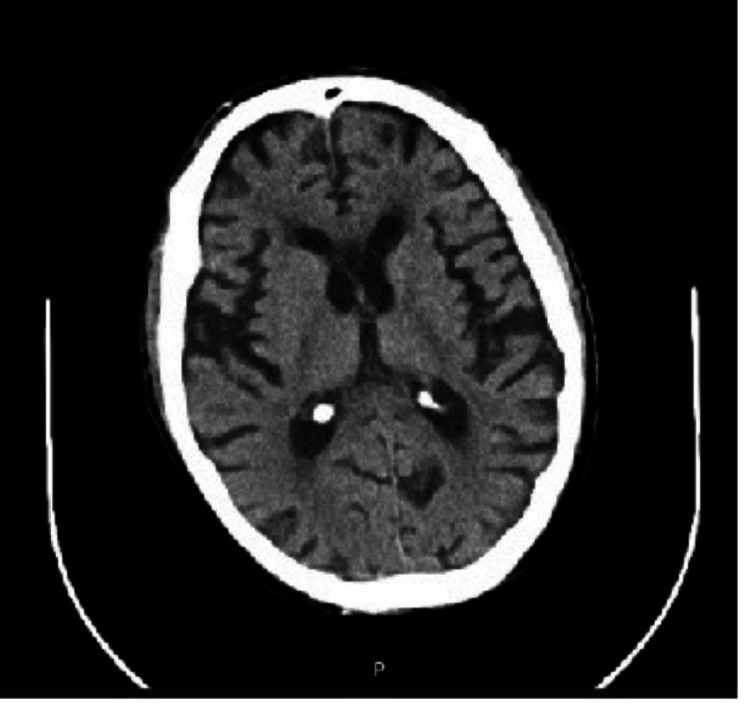
Cranial computed tomography showing generalized parenchymal atrophy and mild periventricular leukoencephalopathy

The chest X-ray showed no consolidations (Figure 2), while the cranial computed tomography (CT) revealed marked generalized parenchymal atrophy and mild periventricular arteriopathic leukoencephalopathy without acute vascular lesions or sequelae (Figure 3).

The lumbar puncture revealed elevated cerebrospinal fluid protein (157 mg/dL) without pleocytosis (Table 2).

**Table 2:** Cytological analysis of cerebrospinal fluid

Cerebrospinal Fluid Biochemistry	Result	Reference Values
Proteins	157 mg/dL	15-40 mg/dL
Glucose	132 mg/dL	40-70 mg/dL
Leukocytes	<3/mm^3^	<3/mm^3^

In this context, the patient was transferred to the intensive care unit (ICU) for closer monitoring and advanced care. Subsequently, infectious serologies returned positive for *Coxiella burnetii* IgG (1:1280) and *Rickettsia conorii *IgM(1:1280). Given the cross-reactivity of *Coxiella burnetii*, phase I and II antibodies were tested and found negative. Thus, HLH secondary to *Rickettsia conorii* infection was confirmed, and doxycycline was added to the treatment regimen, maintaining the previously initiated dexamethasone.

**Figure 4:**
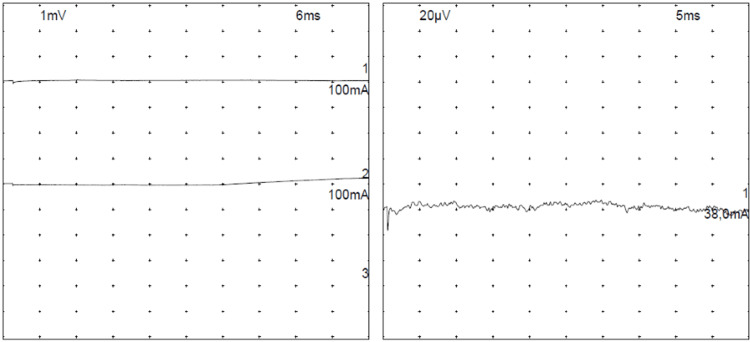
Electromyogram showing sensory-motor axonal polyneuropathy, with loss of voluntary muscle activation

After 17 days in the ICU, the patient was transferred back to the internal medicine ward, where paraplegia and hypoesthesia in the L1-S4 dermatomes were noted. Electromyography (Figure 4) confirmed peripheral sensorimotor axonal polyneuropathy, attributed to HLH.

Additional workup included dorsolumbar magnetic resonance imaging (MRI), which identified diffuse signal changes in the vertebrae of the dorsal and lumbosacral segments. No pathologic contrast enhancements, abnormal tissue masses, or degenerative changes compromising canal or foraminal dimensions were observed. Metabolic, nutritional, and other causes of HLH were excluded.

The patient completed 21 days of doxycycline therapy and, on the 41st day of hospitalization, was discharged to a rehabilitation center. He stayed there for one month and showed complete recovery of neurological deficits.

## Discussion

HLH is a rare and potentially fatal condition characterized by uncontrolled immune hyperactivation. When untreated, it is associated with high mortality rates, making early recognition and immediate intervention crucial for a successful treatment. The broad spectrum of clinical manifestations, nonspecific clinical and laboratory findings, and the rarity of this syndrome contribute to diagnostic delays [8].

In this case, clinical findings such as fever, a maculopapular rash, and pancytopenia led to the diagnosis of spotted fever, consistent with positivity for *Rickettsia conorii* and *Coxiella burnetii*. The positivity for* Coxiella burnetii* was attributed to cross-reactivity. However, the rapid clinical deterioration, accompanied by marked hyperferritinemia, elevated soluble CD25, and hypofibrinogenemia, indicated a hyperinflammatory syndrome, leading to an early diagnosis of secondary HLH.

Diagnosing HLH is challenging due to overlapping manifestations with other clinical conditions [2]. Despite the limited data available, the HLH-2004 diagnostic criteria remain widely used for secondary causes of HLH in adults. At least five of the eight criteria are required to establish a diagnosis. In the presented case, the patient met five of the eight criteria (fever, pancytopenia, hypofibrinogenemia/hypertriglyceridemia, hyperferritinemia, and elevated soluble CD25), supporting the diagnostic hypothesis [4].

Nonetheless, the rarity of the association between *Rickettsia *spp. and HLH increases the risk of diagnostic delay. A review published in February 2024 included 69 articles (totaling 98 patients) and identified only 14 cases (14.3%) of HLH complicated by *Rickettsia *spp. infection [9].

This case emphasizes the importance of considering HLH in the differential diagnosis of patients with *Rickettsia *spp. infections and laboratory findings suggestive of immune hyperactivation.

Neurological manifestations are an important yet often underappreciated component of HLH. They vary and, when present, typically occur 14 to 21 days after infection. They include seizures, gait ataxia, dysmetria, dysarthria, and changes in consciousness and/or behavior, as observed in this patient. These findings may dominate the clinical picture or occur alongside other signs or symptoms [10].

The central nervous system (CNS) is the most affected site (in 10% of cases), significantly worsening the prognosis and potentially causing permanent sequelae. CNS involvement results from activated macrophage infiltration, producing symptoms like encephalitis. Brain MRI findings include hypodense areas or necrosis, and cerebrospinal fluid alterations are present in 50% of cases, typically showing mononuclear pleocytosis (lymphocytic meningitis) or albuminocytologic dissociation [11].

Peripheral axonal neuropathy due to* Rickettsia conorii *infections is rare, typically presenting as subacute, often asymmetrical, and involving mixed sensory and motor fibers [12]. The pathophysiology remains unclear but likely involves a multifactorial process, including vascular invasion by the pathogen and an immune response contributing to neuronal degeneration [12].

Three cases of peripheral polyneuropathy associated with *Rickettsia conorii *infection have been documented, all serologically confirmed as in the present case. In the first case, a 78-year-old woman developed sensory ataxic neuropathy 17 days post-infection, treated with doxycycline but with only partial deficit improvement [13]. Another case involved a 63-year-old woman who developed acute demyelinating motor-sensory neuropathy, consistent with Guillain-Barré syndrome, treated with doxycycline, ceftriaxone, and immunoglobulin, but progressed to chronic inflammatory demyelinating polyneuropathy [14]. Finally, a 76-year-old man experienced acute facial paralysis followed by flaccid tetraparesis one week after infection, treated with two weeks of doxycycline with complete recovery [12].

It is essential to exclude neoplasms, rheumatic diseases, immunodeficiencies, or infections, as these conditions are common in HLH patients, especially adults. Treatment aims to suppress inflammation as early as possible [15].

The EULAR 2022 guidelines recommend early empirical immunomodulatory therapy for HLH, alongside supportive care and targeted antimicrobial/antifungal/antiviral or antiparasitic therapy based on the suspected microorganism. High-dose corticosteroids (oral prednisone or IV methylprednisolone, 1-2 mg/kg/day or IV dexamethasone, 10 mg/m^2^/day), immunoglobulins (1 g/kg/day for the first two days, then 0.4-1 g/kg/day for two to five days), or anakinra (5-10 mg/kg/day), an interleukin-1 blocker, are suggested for severe infections with persistent symptoms and/or multiorgan dysfunction [16].

The NHS England recommends anakinra for patients who do not respond to or have contraindications for conventional immunosuppressive therapy, as it has shown efficacy in reducing mortality and improving inflammatory parameters [17]. Thus, the choice of immunosuppressant should always be individualized to the patient's clinical condition [16,17]. In 2004, these guidelines were updated, combining etoposide, dexamethasone, and cyclosporine as initial therapy (one to eight weeks) for patients with HLH regardless of genetic or familial evidence [4].

## Conclusions

HLH is a rare, potentially fatal condition that presents significant challenges in clinical practice, particularly due to the nonspecific nature of its early signs and symptoms. This case underscores the importance of early recognition, especially in the presence of persistent fever, pancytopenia, and significant inflammatory alterations such as hyperferritinemia. A multidisciplinary approach was essential in managing both systemic and neurological complications, including the rare manifestation of peripheral axonal neuropathy associated with *Rickettsia *spp. infections but potentially reversible with timely diagnosis and treatment.

The favorable outcome in this case highlights the positive impact of early, tailored, and targeted interventions in reducing morbidity and mortality and ensuring complete functional recovery. Furthermore, the case contributes to raising awareness of the rare manifestations of HLH, particularly in adults, when associated with *Rickettsia *spp. infections. Early detection and integrated management were critical in modifying the clinical course of this syndrome, preventing severe complications and offering the patient the possibility of a full recovery and improved quality of life.
